# Noise increases the correspondence between artificial and human vision

**DOI:** 10.1371/journal.pbio.3001477

**Published:** 2021-12-10

**Authors:** Jessica A. F. Thompson

**Affiliations:** Human Information Processing Lab, Department of Experimental Psychology, University of Oxford, Oxford, United Kingdom

## Abstract

This Primer explores the implications of a recent PLOS Biology study, arguing that noise-robustness, a property of human vision that standard computer vision models fail to mimic, provides an opportunity to probe the neural mechanisms underlying visual object recognition and refine computational models of the ventral visual stream.

## Teaser

The best performing computer vision systems are based on deep neural networks (DNNs). A study in this issue of *PLOS Biology* shows that DNNs trained on noisy stimuli are better than standard DNNs at mirroring both human behavioral and neural visual responses.

Core visual object recognition refers to the rapid recognition of the identity of an object during a single fixation. In humans and other primates, since this process happens very quickly, it is thought to be the result of primarily feedforward neural processing in the ventral visual stream [1]. Today, the best performing computer vision systems are based on deep neural networks (DNNs), which often achieve or even surpass human performance on object recognition tasks. DNNs are currently championed as models of the neural processing underlying human object recognition, based on an observed correspondence between patterns of activity in DNNs and neural activity throughout the ventral visual stream [2]. However, the full multiplicity of human vision is not well captured by a single accuracy value. More nuanced characterization of visual behavior is needed. Constructive research in this area probes the source not just of correspondences but also the various divergences between of human and machine vision. In this issue of *PLOS Biology*, Jang and colleagues [3] explore one such divergence: noise robustness.

Standard datasets for training DNNs to recognize objects often consist of relatively clean and clear photos of objects. Networks trained on such datasets will show deficits in viewing conditions that were not included in the training data, for example, in different lighting or weather. Data augmentation, which consists of applying various transformations to the training images to artificially increasing the size and variety of a dataset, is commonly used to improve a network’s ability to generalize [4].

Jang and colleagues explored 2 of such transformations, the addition of (1) Gaussian pixelated (spatially uncorrelated) noise and (2) Fourier scrambled (spatially correlated) noise. These controversial stimuli, which easily confuse standard trained DNNs, are used to arbitrate among candidate computational models and to probe the mechanisms underlying noise robustness in human vision. The authors found that DNNs were more severely disrupted by Gaussian pixelated noise whereas humans were more disrupted by Fourier scrambled noise. Networks that received additional training on images with noise added to them displayed more human-like behavior. Functional neuroimaging revealed that noise training also increased the correspondence to human brain activity through the ventral visual stream.

**Fig 1:**
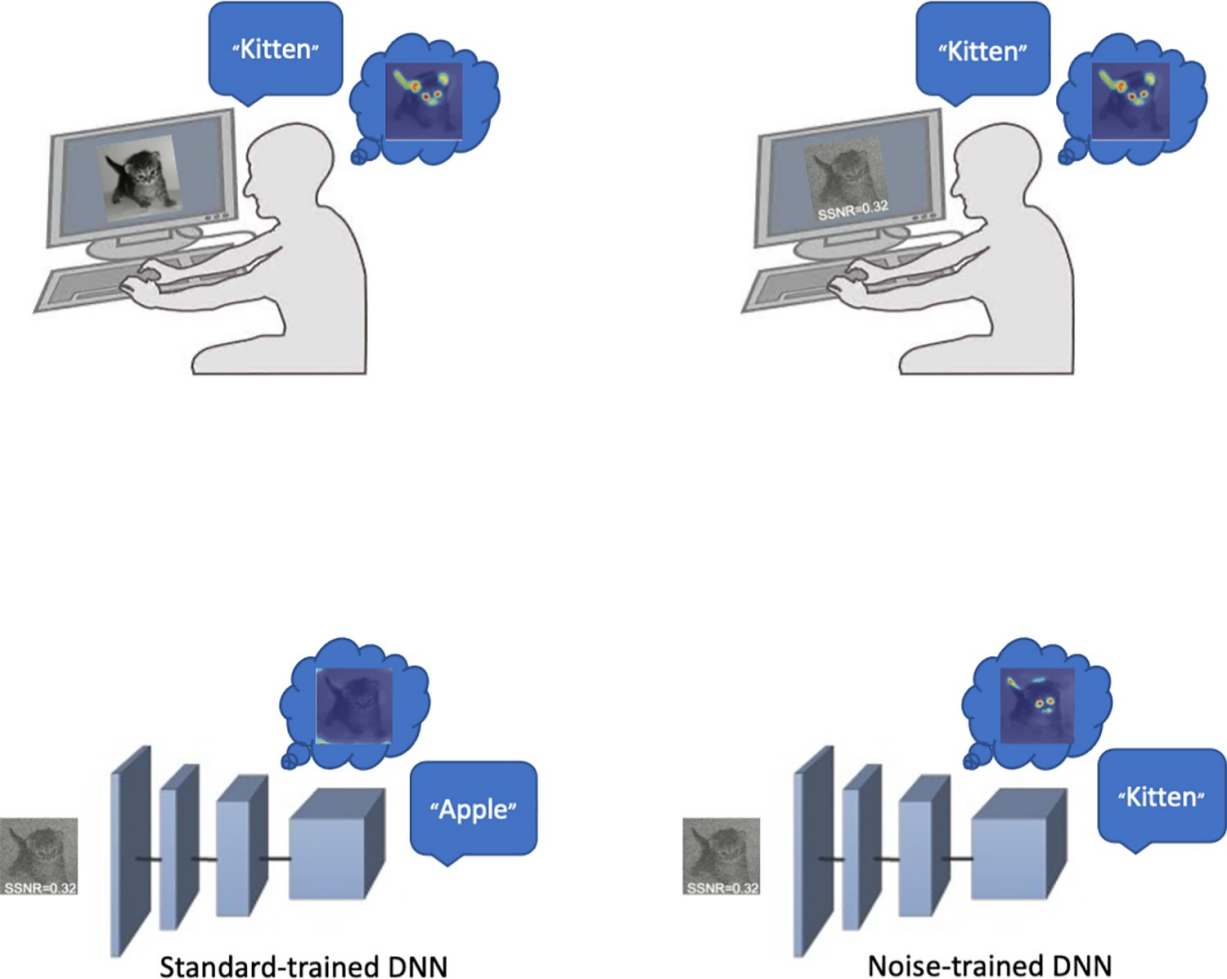
Noise-robust vision in humans and machines. Human visual object recognition is robust to various kinds of noise. DNNs trained according to standard procedures are significantly less robust to noise. However, fine-tuning with noisy images not only makes DNNs more robust; it also brings the behavior and activity of the network into greater alignment with the human visual system. DNN, deep neural network; SSNR, signal-to-signal-plus-noise ratio.

By parametrically varying the amount of noise added to the image stimuli, Jang and colleagues quantified performance on the object recognition task as a function of the signal-to-signal-plus-noise ratio (SSNR). This allowed for the calculation of recognition thresholds for both human viewers and the computer vision models. The behavior of both human and machine viewers was also characterized with relevance maps. For the DNNs, these heatmaps show the regions of the image that are most diagnostic of the network’s classification. Human viewers were asked to “paint” the regions of the image that were most informative for their decision. Examples of these relevance maps can be seen in Fig 1. Both recognition thresholds and relevance maps were more human-like for noise-robust DNNs [3].

This research extends previous work showing that DNNs are severely affected by various image corruptions and that the patterns of errors they make on such images do not mirror the mistakes that humans make [5]. The SSNR threshold for the noise-trained DNN reported by Jang and colleagues was slightly lower than that of the human viewers [3]. This is in line with previous work, which found that data augmentation can lead to superhuman performance on the specific image corruptions seen during training [6]. Rusak and colleagues [7] also demonstrated that careful noise training can help DNNs generalize to unseen image corruptions as well. The neuroimaging results presented by Jang and colleagues [3] provides novel evidence that noise training brings the network’s internal information processing, not just its output, into greater alignment with that of the human visual system.

What does this body of research say about the mechanisms underlying human noise robustness? Jang and colleagues speculate that robustness to visual noise is acquired, at least in part, through learning and experience, but the exact mechanisms by which visual experience imparts robustness remains an open question. Jang and colleagues showed that their noise training procedure allowed the network to generalize to natural weather conditions. The hypothesis implied by these results is that simple exposure to a variety of viewing conditions will generalize to conditions that share statistical properties. However, Geirhos and colleagues [5] conclude that humans and DNNs generalize in fundamentally different ways. Their analysis, which included many different types of image transformations, found transformations that appear very similar to human viewers but which did not enable generalization in DNNs (networks trained on one do not generalize to the other). There are likely additional inductive biases that influence human generalization to corrupted images that are not captured in current DNN models and training algorithms. Since Jang and colleagues only investigated 2 types of noise, their experiments are less well suited to address the generalization question.

The comparison of human and machine perception is fraught with challenging complications. Funke and colleagues highlight how human bias can affect the interpretation of results, the challenge of aligning the experimental conditions between human and machine viewers, and the importance of distinguishing between necessary and sufficient conditions [8]. As this is a new area of study, the methodological and interpretive norms are still being established. For example, several authors have questioned the validity of attribution methods, including the method used by Jang and colleagues to produce their relevance maps [9,10]. However, the desiderata for methods used in machine learning research may be different than those for comparison between human and machine perception. The fact that these relevance maps showed some alignment with the human diagnostic regions may provide indirect evidence for their bearing on human perception.

DNNs persist as the best model of human visual object recognition despite growing documentation of the ways in which they deviate from human behavior and neural activity. These deviations do not necessarily provide cause for the rejection of such models. Rather, they provide useful signals for their refinement. For example, recently, Xu and Vaziri-Pashkam published a thorough comparison of 14 DNNs to activity throughout the human visual system. They found that although early visual regions were well captured by the activity of early network layers, significant variance was left unaccounted for in high-level visual areas [11]. The comparison in Jang and colleagues found that noise training increased the brain-model correspondence particularly at higher-level visual areas [3]. Thus, together, these results point to candidate model refinements to ultimately build better models of the neural information processing underlying human vision.
